# Biphasic decay of intact SHIV genomes following initiation of antiretroviral therapy complicates analysis of interventions targeting the reservoir

**DOI:** 10.1073/pnas.2313209120

**Published:** 2023-10-16

**Authors:** Mithra R. Kumar, Emily J. Fray, Alexandra M. Bender, Carolin Zitzmann, Ruy M. Ribeiro, Alan S. Perelson, Dan H. Barouch, Janet D. Siliciano, Robert F. Siliciano

**Affiliations:** ^a^Department of Medicine, Johns Hopkins University School of Medicine, Baltimore, MD 21205; ^b^Los Alamos National Laboratory, Los Alamos, NM 87545; ^c^Center for Virology and Vaccine Research, Beth Israel Deaconess Medical Center, Boston, MA 02215; ^d^HHMI, Baltimore, MD 21205

**Keywords:** HIV, latent reservoir, IPDA, SHIV

## Abstract

The latent reservoir for HIV-1 in resting CD4^+^ T cells persists despite antiretroviral therapy (ART) and precludes cure. Reservoir-targeting interventions are evaluated in ART-treated macaques infected with simian immunodeficiency virus (SIV) or simian-human immunodeficiency virus (SHIV). Efficacy is determined by reservoir measurements before and after the intervention. However, most proviruses persisting in the setting of ART are defective. In addition, intact HIV-1 and SIV genomes undergo complex, multiphasic decay observable when new infection events are blocked by ART. Intervention-induced elimination of latently infected cells must be distinguished from natural decay. Here, we address these issues for SHIV. We describe an intact proviral DNA assay that allows digital counting of SHIV genomes lacking common fatal defects. We show that intact SHIV genomes in circulating CD4^+^ T cells undergo biphasic decay during the first year of ART, with a rapid first phase (t_1/2_ = 30.1 d) and a slower second phase (t_1/2_ = 8.1 mo) that is still more rapid that the slow decay observed in people with HIV-1 on long-term ART (t_1/2_ = 3.7 y). In SHIV models, most interventions are tested during 2nd phase decay. Natural 2nd phase decay must be considered in evaluating interventions as most infected cells present at this time do not become part of the stable reservoir. In addition, for interventions tested during 2nd phase decay, a caveat is that the intervention may not be equally effective in people with HIV on long-term ART whose reservoirs are dominated by latently infected cells with a slower decay rate.

## Significance

Persistence of a latent reservoir in resting CD4^+^ T cells is the major barrier to curing HIV-1 infection. Non-human primates infected with simian–human immunodeficiency virus (SHIV) provide a valuable model for cure strategies. As with HIV-1, most SHIV proviruses persisting in animals treated with antiretroviral therapy (ART) are defective and irrelevant to cure. The lack of assays that selectively quantify intact proviruses impeded understanding of reservoir dynamics in this model. Here, we describe an assay for intact SHIV proviruses and use it to define rapid biphasic decay processes during the first year of ART when many interventions are tested. This study provides insights into early decay kinetics of intact SHIV genomes that must be considered in evaluating cure interventions.

Combination antiretroviral therapy (ART) effectively inhibits HIV-1 replication, preventing infection of susceptible cells and reducing viremia to below the limit of detection of clinical assays (1–3). However, ART alone is not curative, as viremia typically rebounds within weeks of ART discontinuation (4–7). Rebound viremia is attributed to virus production from persistent viral reservoirs that persist despite ART and antiviral immune responses. The best-characterized reservoir is a small pool of latently infected resting CD4^+^ T cells. This reservoir was initially identified using the Quantitative Viral Outgrowth Assay (QVOA), which measures the frequency of latently infected cells capable of producing replication-competent virus following maximal T cell stimulation (8–12). Longitudinal studies using the QVOA in people with HIV (PWH) on suppressive ART revealed that the half-life of the latent reservoir in resting CD4^+^ T cells is 44 mo, long enough to ensure lifelong persistence of the infection even with optimal ART (13, 14). Recently, it has become clear that the reservoir does not continue to decay at this rate in PWH on very long-term ART (15). Rather, after several years, the decay slows, and the size of the reservoir may actually begin to increase due to infected cell proliferation. Consequently, therapeutic interventions targeting the latent reservoir are necessary for a cure. Their development depends on expanding our current understanding of reservoir dynamics.

Non-human primates (NHPs), especially Indian-origin and Chinese-origin rhesus macaques, infected with simian immunodeficiency virus (SIV) are important animal models that emulate key features of HIV-1 infection, including high viral loads (16), progressive CD4^+^ T cell depletion (17–19), envelope protein (Env) trafficking and evolution (20, 21), response to ART (16, 22–24), and establishment of a latent reservoir in resting CD4^+^ T cells (25–27). Although studies of NHPs infected with various forms of SIV have aided in the analysis of cure strategies, structural differences between the SIV and HIV-1 Env proteins restrict application of this model in studies of interventions targeting HIV-1 Env. This limitation has been overcome by infecting NHPs with chimeric simian–human immunodeficiency virus (SHIV) in which the SIV *env* gene has been replaced with one from an HIV-1 isolate (28–32). SHIV models have been used extensively to investigate vaccines, broadly neutralizing antibodies, and other Env-targeting interventions (33–39). The widely used SHIV_SF162P3_ was derived from SIV_mac239_, by replacing the SIV *tat*, *rev*, and *env* genes with the corresponding sequences from HIV-1_SF162_ (29, 32).

The utility of NHP models for preclinical studies of the latent reservoir and HIV-1 cure strategies depends on the similarities and differences between reservoir dynamics in SIV, SHIV, and HIV-1 infections and on accurate assays to quantify the latent reservoir before and after therapeutic interventions. Due to the cost of maintaining animals on long-term ART, most cure studies in NHP models involve animals that have been on ART for relatively short periods of time (<2 y). It is therefore important to understand reservoir dynamics in the months immediately following initiation of ART. When new infection events are blocked by ART, the decay of populations of cells that were infected prior to ART can be observed. Complex decay patterns observed in HIV-1 (1, 13–15, 40–47), and SIV (16, 48) infections indicate the presence of multiple distinct populations of infected cells, and it is important to understand their roles in viral persistence.

Accurate analysis of reservoir dynamics requires the use of assays that selectively detect intact viral genomes. The vast majority of HIV-1 proviruses persisting during ART are defective (49–57). Recent studies have shown that proviruses with the same types of fatal defects also persist in macaques that have been infected with SIV or SHIV and treated with ART (48, 58). The observed defects include large internal deletions and APOBEC3-mediated hypermutation. These defects typically affect multiple viral genes, and, consequently, defective proviruses do not contribute to viral rebound upon ART interruption (57). Therefore, proviruses with large deletions and extensive hypermutation should be excluded from reservoir measurements. While the QVOA only detects replication-competent proviruses, it is impractical for kinetic studies due to the large blood volumes required and the failure of the assay to detect proviruses that are not induced by a single round of T cell activation (49, 59, 60). We have therefore developed multiplex droplet digital PCR (ddPCR) assays that selectively quantify proviruses that lack large internal deletions and hypermutation (57, 58). Compared with widely used single amplicon PCR assays (61), these multiplex ddPCR assays provide a much better estimate of the frequency of genetically intact proviruses with the potential to cause viral rebound upon ART interruption. They have therefore been termed intact proviral DNA assays (IPDAs). These assays have provided insights into reservoir dynamics in treated PWH on ART (15, 46, 47, 57, 60, 62–65) and in SIV-infected macaques on ART (48, 58).

Here, we use a SHIV IPDA to describe the decay of intact SHIV proviruses during the first year of ART. The decay kinetics are compared to those of HIV-1 and SIV. Our data indicate that understanding the natural decay of SHIV-infected cells is important for the correct interpretation of cure trial results in SHIV models.

## Results

### A ddPCR-based Assay to Measure Intact SHIV Genomes.

**Fig. 1.**
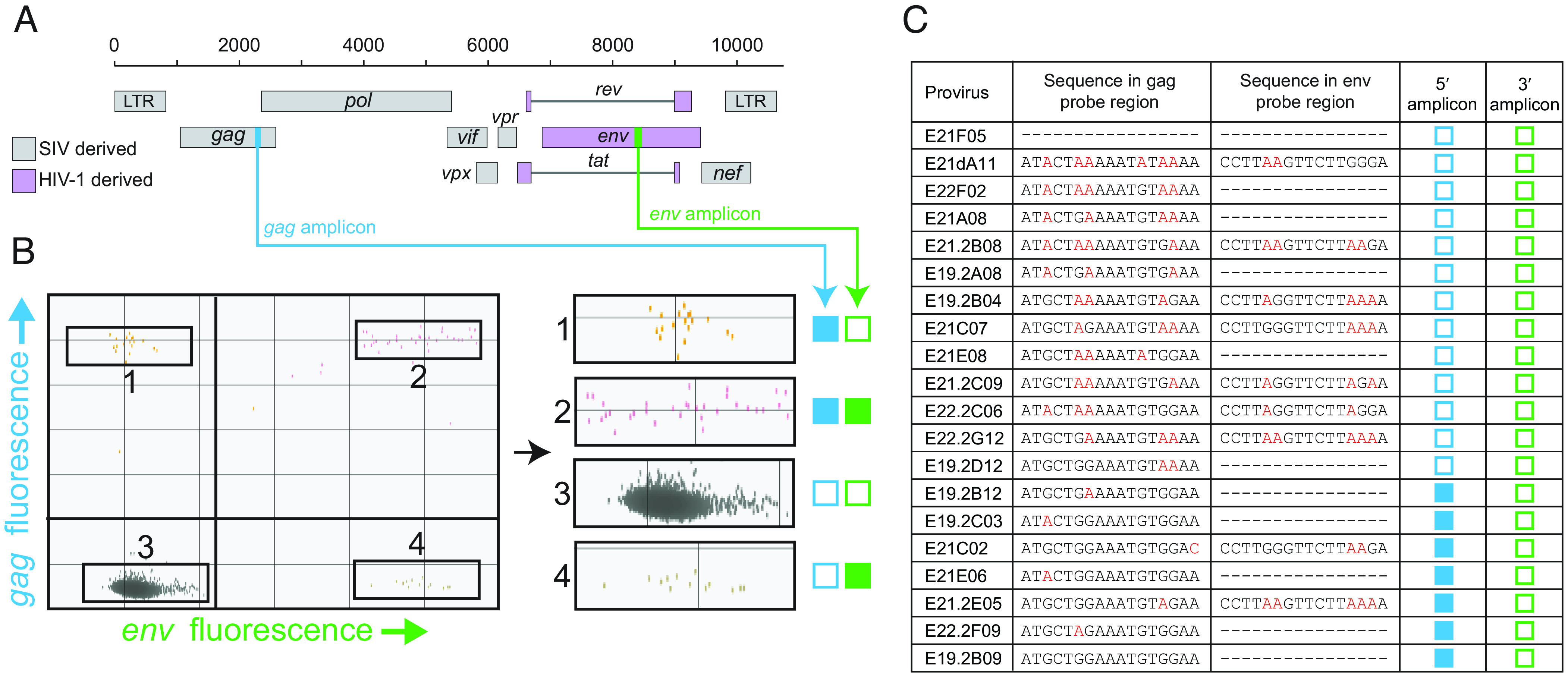
Design of a ddPCR assay selective for intact SHIV genomes. (*A*) Map of the genome of SHIV_SF162P3_ indicating the regions derived from SIV (gray) and HIV-1 (purple) and the positions of the SHIV IPDA *gag* (blue) and *env* (green) amplicons. (*B*) Representative IPDA dot plot from a SHIV_SF162P3_-infected macaque on ART showing for individual droplets the level of fluorescence from the *gag* and *env* amplicons. The indicated regions of the dot plot are enlarged on the right to show individual droplets that are single positive for the *gag* amplicon (1), double positive (2), double negative (3), and single positive for the *env* amplicon (4). Blue and green boxes indicate successful (filled) or unsuccessful (empty) amplification for the *gag* and *env* amplicons, respectively. Intact genomes are digitally counted as double-positive droplets (quadrant 2). The vast majority of droplets lack a SHIV genome and are also positioned in quadrant 3. (*C*) Sequences of hypermutated SHIV genomes in the regions of the *gag* and *env* probes in proviruses from infected macaques (58). Dashes indicate deletion of the relevant regions. Mismatches with the probe sequence due to hypermutation are indicated in red. Expected IPDA results for the *gag* and *env* amplicons are shown the blue and green boxes, respectively. Two or more mismatches generally prevent amplification (open boxes) (57). Note that none of the hypermutated genomes shown here are double positive.

Accurate measurement of the decay kinetics of SHIV genomes requires a precise assay that can be run on small blood samples and that selectively captures intact viral genomes. We have previously shown that relative to standard single amplicon PCR assays, improved selectivity for intact HIV-1 and SIV proviruses can be achieved by simultaneously interrogating individual proviruses at two appropriately spaced positions using ddPCR (57, 58). Most proviral deletions are large, on average encompassing half of the genome (49, 51, 57). Therefore, requiring amplification from two different, adequately spaced regions allows good discrimination between intact and deleted viral genomes (57, 58). In addition, if the amplicon probes are positioned at sites containing APOBEC3 target sequences, hypermutated proviruses can also be distinguished by including in the ddPCR reactions unlabeled competition probes with G-to-A mutations (48, 57, 58). Using these principles, we designed an IPDA for SHIV proviruses (Fig. 1). Because most SHIVs used in HIV-1 vaccine and cure research contain the HIV-1 *env* gene, we used the *env* amplicon from the HIV-1 IPDA which covers a conserved region in the Rev Response Element (Fig. 1*A*). Approximately three quarters of SHIV genomes have large deletions affecting the 3′ end of the genome (58), and therefore many deleted proviruses will be negative for this amplicon. In addition, the probe site for this amplicon has two TGGG motifs that are frequently mutated by APOBEC3 enzymes, allowing additional discrimination between intact and hypermutated proviruses (57, 58). The labeled *env* probe does not bind well if 2 or more of the Gs in the target genome are mutated, and, in addition, the reaction includes an unlabeled competitor probe that binds specifically to hypermutated sequences in this region. Thus, most hypermutated genomes will fail to give a signal for the *env* amplicon (57).

Using previously published near full genome sequences of SHIV proviruses (58), we then identified the optimal positioning of a 5′ amplicon. While the HIV-1 and SIV IPDAs use 5′ amplicons in the packaging signal (Ψ) and *pol* regions, respectively (57, 58), we found that optimal discrimination between intact and defective SHIV proviruses could be achieved with a 5′ amplicon in a region of the *gag* gene that included sites frequently hypermutated in SHIV proviruses (Fig. 1 *B* and *C*). As is the case with the *env* amplicon, 2 or more G-to-A mutations in the probe site for the *gag* amplicon will prevent binding of the labeled probe. To provide for even more stringent exclusion of hypermutated sequences, we also include in the reactions unlabeled hydrolysis probes that bind competitively to hypermutated proviruses in this region (*Methods*). Analysis of samples from SHIV-infected macaques on ART with this IPDA revealed numerous defective proviruses that gave amplification at only the *gag* or *env* sites (Fig. 1*B*). In addition, proviruses with extensive hypermutation do not give amplification at either amplicon position (Fig. 1*C*). These double-negative proviruses cannot be directly counted because they are partitioned into the lower left quadrant (Q3) along with the excess of droplets that do not contain a provirus (Fig. 1 *B* and *C*). The same is true for proviruses with large deletions encompassing both amplicons. Thus, this assay does not allow for accurate counting of all defective proviruses. Nevertheless, the assay does provide for precise digital counting of the proviruses that lack the most common fatal defects, large deletions and hypermutation. For simplicity, these proviruses are termed intact, with the understanding that some double-positive proviruses may have minor defects (57).

Using plasmid and synthetic double-stranded DNA templates (gBlocks) as controls, we optimized the thermal-cycling conditions and verified the specificity and sensitivity of the SHIV IPDA as previously described (48). As DNA shearing between the IPDA amplicons during DNA isolation reduces the number of double-positive droplets (57), we developed a second multiplexed ddPCR reaction with amplicons in the host gene RPP30 (*SI Appendix*, Fig. S1*A*). The two primer/probe sets in the RPP30 gene are spaced at the same distance as the *gag* and *env* IPDA amplicons (~6,200 bp), and therefore the ratio of RPP30 single-positive to double-positive droplets measured using this assay allows for the calculation of a DNA shearing index (DSI) which can be used in downstream analysis to correct for shearing-related reductions in the double-positive droplet count as previously described (57). The data from the RPP30 assay can also be used to calculate the input cell equivalents for each sample, which are used to determine the frequency of intact proviruses per million cells. The mean DSI across the 112 samples from SHIV-infected macaques was 0.303 ± 0.044 (*SI Appendix*, Fig. S1*B*). Thus, only ~30% of full-length SHIV proviruses would undergo shearing between the amplicons during the DNA extraction and ddPCR steps. This value is within the range for which corrections can be successfully applied (57).

Another consideration in the measurement of intact SHIV genomes is the presence of 2LTR circles which are generated when completed reverse transcripts undergo end-to-end joining rather than integration. These replication-defective forms are more stable than linear unintegrated forms of viral genomes and can complicate PCR-based measurement of intact proviruses (47, 48, 58, 66–70). They frequently contain one or both regions interrogated by the SHIV IPDA and can affect enumeration of intact proviruses (48, 58). Therefore, our analysis includes a third multiplexed ddPCR assay that combines an amplicon spanning the SIV 2LTR junction (70) and the SHIV IPDA *env* amplicon. This allows us to digitally count 2LTR circles containing the *env* gene. These are then subtracted from the IPDA double-positive counts. Based on the results of these three assays, the DSI-corrected frequency of intact SHIV genomes per million input cells are reported (see *Methods* for details).

It is also important to note that the assay does not determine whether the viral genomes detected are integrated into host cell DNA. However, as discussed above, 2LTR circles are separately enumerated, and linear unintegrated forms are labile (t_1/2_ ~ 2 d) (40, 44). Therefore, we generally use the term “genome” when discussing analysis of samples taken before or shortly after initiation of ART and the term “provirus” when discussing analysis of samples taken months after ART initiation.

### SHIV Dynamics in ART-Treated Macaques.

**Fig. 2.**
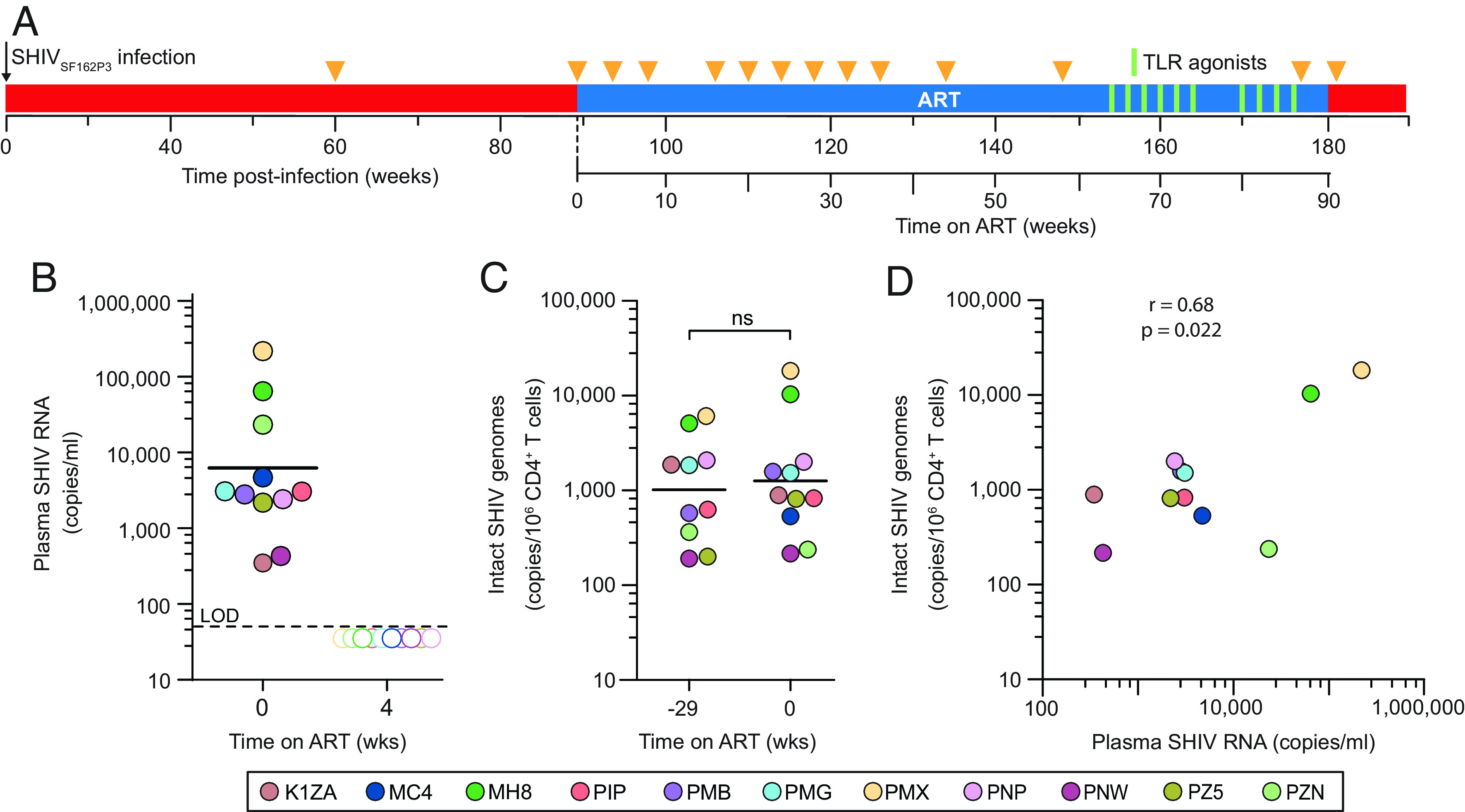
Set point levels of viremia and intact SHIV genomes. (*A*) Timeline for SHIV_SF162P3_ infection. Animals were infected by repeated intrarectal inoculations with SHIV_SF162P3_ and after 89 wk were treated with ART for 90 wk (blue bar). Sampling times are indicated by the orange triangles. Seven of the animals were given 10 doses of TLR7 or TLR8 agonists between weeks 64 and 86 of ART (green bars) as described in text. (*B*) Decay of plasma SHIV RNA following initiation of ART. Baseline levels (week 0 of ART) are shown for individual animals. The geometric mean value is indicated by the black line. By week 4, plasma SHIV RNA levels were below the limit of detection (LOD, 50 copies/mL) in all animals (open symbols). (*C*) Steady-state pre-ART levels of intact viral genomes measured by IPDA at 60 wk post infection (−29 wk relative to the start of ART) and 89 wk post infection (time 0, day of ART initiation). Values are geometric means of 3 replicate determinations of intact genomes per 10^6^ peripheral blood CD4^+^ T cells, corrected for DNA shearing and env^+^ 2LTR circles. Week −29 value for MC4 not available. (*D*) Correlation between plasma virus levels and the frequency of intact SHIV genomes in circulating CD4^+^ T cells at the time of ART initiation.

We used the SHIV IPDA to investigate reservoir dynamics in 11 SHIV_SF162P3_-infected rhesus macaques that were treated with ART starting at 89 wk post infection (Fig. 2*A*). Importantly, initiation of ART after set point viremia is established provides a realistic model of ART in PWH, most of whom start therapy during chronic infection. At the time of ART initiation, levels of plasma SHIV RNA in different animals varied between 413 and 218,640 copies/mL (geometric mean = 4,814 copies/mL, Fig. 2*B*). Using the IPDA, we measured the frequency of intact SHIV genomes in peripheral blood CD4^+^ T cells at two different time points prior to ART. Interestingly, the geometric mean frequencies of intact genomes at 60 wk post-infection (1,013 copies per million CD4^+^ T cells) and at 89 wk post-infection (1,279 copies per million CD4^+^ T cells) were not significantly different (*P* = 0.43), consistent with a relatively stable set point level of infected cells in untreated infection (Fig. 2*C*). At the time of ART initiation (89 wk post infection), the frequency of intact SHIV genomes was strongly correlated (r = 0.68, *P* = 0.022) with the level of plasma virus (Fig. 2*D*). Many of these viral genomes are likely to be from recently infected cells that are propagating the infection prior to ART, and some may be labile linear unintegrated forms (40, 44). The frequency of CD4^+^ T cells with intact genomes in untreated SHIV infection (1,279 copies per million CD4^+^ T cells) is comparable to that observed in PWH prior to treatment (2,255 copies per million CD4^+^ T cells), but it is lower than that observed in untreated SIV_mac251_-infected macaques (5,036 copies per million CD4^+^ T cells) (47, 48, 58). These differences are generally consistent with differences in set point levels of viremia in SHIV, HIV-1, and SIV infections.

By 4 wk after initiation of ART, plasma levels of SHIV RNA had fallen below the limit of detection (50 copies/mL) in all animals (Fig. 2*B*). For the animal with the highest level of viremia (PMX), this decay represents a decrease of over 3 logs in 4 wk, corresponding to a half-life of <3 d. These results are consistent with the rapid decay of plasma virus observed in HIV-1 and SIV infection when new infection events are blocked by ART (1, 16, 22, 24, 41, 42, 47, 48, 69–73). Thus, ART effectively blocked SHIV replication in these animals.

**Fig. 3.**
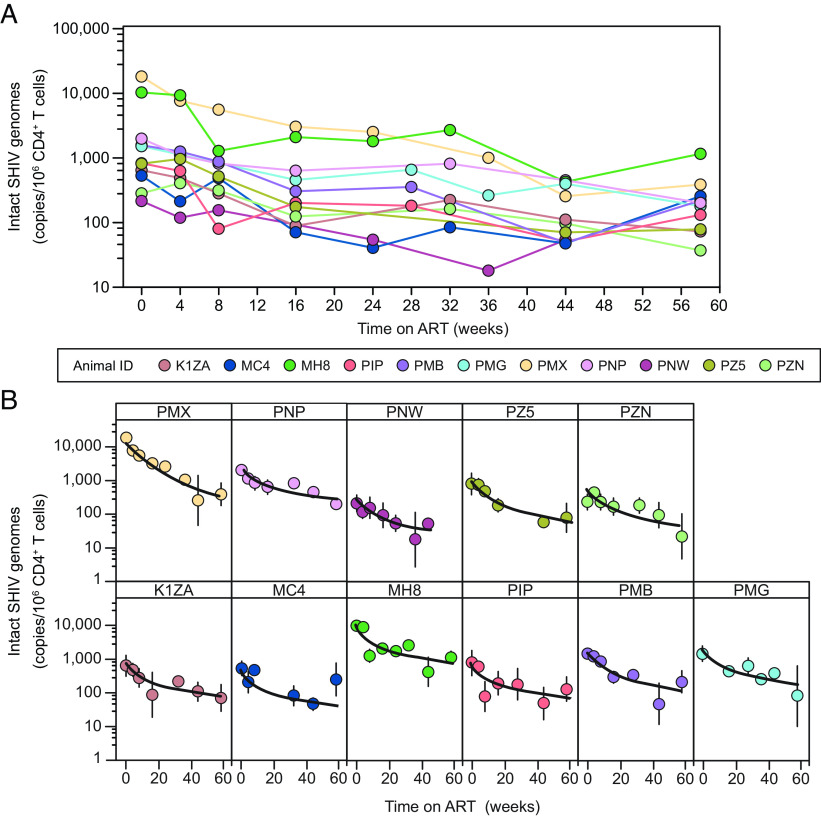
Decay of intact SHIV genomes in peripheral blood CD4^+^ T cells following initiation of ART. (*A*) Frequencies of intact SHIV genomes as detected by IPDA in circulating CD4^+^ T cells following initiation of ART. (*B*) Frequencies in individual animals fit by a biexponential decay model. Thick black lines indicate model fits for individual animals. See *SI Appendix*, Table S2 for parameter estimates for each animal. Vertical bars indicate 95% CIs for individual time points, based on assay triplicates.

By performing longitudinal IPDA measurements over the first year of ART in SHIV_SF162P3_-infected macaques, we examined the decay rate of intact SHIV genomes when new infection events were blocked by ART. Consistent with recent studies on early HIV-1 and SIV decay dynamics (47, 48), we found that in circulating CD4^+^ T cells, intact SHIV genomes also followed a biphasic early decay pattern (Fig. 3 *A* and *B*). After relatively rapid decay during the first few weeks of ART, the frequency of intact genomes declined more slowly for the rest of the observation period. To confirm that the decay of intact genomes occurred in multiple phases, we used a non-linear mixed-effect approach to fit the decay of intact SHIV genomes to a biexponential decay model (Fig. 3*B* and *SI Appendix*, Tables S1 and S2). Intact genomes decayed with an initial population estimated t_1/2_ of 30.1 d or 4.3 wk (95% CI: 1.6 to 15 wk). Approximately 70% of the intact SHIV genomes present at the time of ART initiation decayed at this relatively rapid rate (*SI Appendix*, Tables S1). Thus, most of the infected cells present at the time of ART initiation do not become part of the stable reservoir because of this rapid decay. Although rapid, this decay is considerably slower than the decay of viremia in the same animals (t_1/2_ < 3 d, Fig. 2*B*), suggesting that plasma virus is mainly produced by infected cells that are not in the circulation and are therefore not detected in IPDA analysis of circulating CD4^+^ T cells (*Discussion*). The IPDA does not determine whether the viral genomes detected are integrated into host DNA, and it is possible that some of the initial decay is due to linear unintegrated viral genomes which are unstable (t_1/2_ ~ 2 d) (40, 44). A correction for 2LTR circles is made as part of SHIV IPDA analysis, and the reported decay of intact genomes does not include 2LTR circles, which are measured separately (see below).

Following the initial decline, SHIV proviruses decayed more slowly with a population t_1/2_ of 35 wk (95% CI: 12 to 108 wk, *SI Appendix*, Table S1). Repeated measurements during the first year of ART revealed differences in the 2nd phase decay rate in different animals (Fig. 3*B* and *SI Appendix*, Table S2). For example, 2nd phase decay was relatively rapid in animal PMX (t_1/2_ = 17.4 wk) and much slower in animal PNP (t_1/2_ = 42.7 wk). Intra-individual variation in the 2nd phase decay of intact proviruses has also been observed in HIV-1 infection (47). The 2nd phase decay rate is not clearly related to viral set point but might reflect immune parameters such as the SHIV-specific CTL response. As discussed below, establishing the trajectory of natural 2nd phase decay by multiple measurements in each individual allows a more accurate assessment of the efficacy of reservoir-targeting interventions carried out during this time interval. After 60 wk of ART, the geometric mean frequency of intact proviruses had fallen by almost 1 log to a value of 151 copies per million CD4^+^ T cells. This result further emphasizes that most infected cells present at the time of ART initiation do not become part of stable reservoir. Importantly, the 2nd phase decay (t_1/2_ = 35 wk) is considerably faster than the quasi-stable third phase decay rate of the latent reservoir in PWH on ART (t_1/2_ = 44 mo) (13, 14). Due to the expense of maintaining macaques on long-term ART, a 3rd phase with a slower decay rate similar to that seen in PWH on long-term ART has not yet been defined for SHIV. In SIV-infected macaques, this quasi-stable 3rd phase did not become apparent until after 2 y of ART (48).

### Dynamics of 2LTR Circles.

**Fig. 4.**
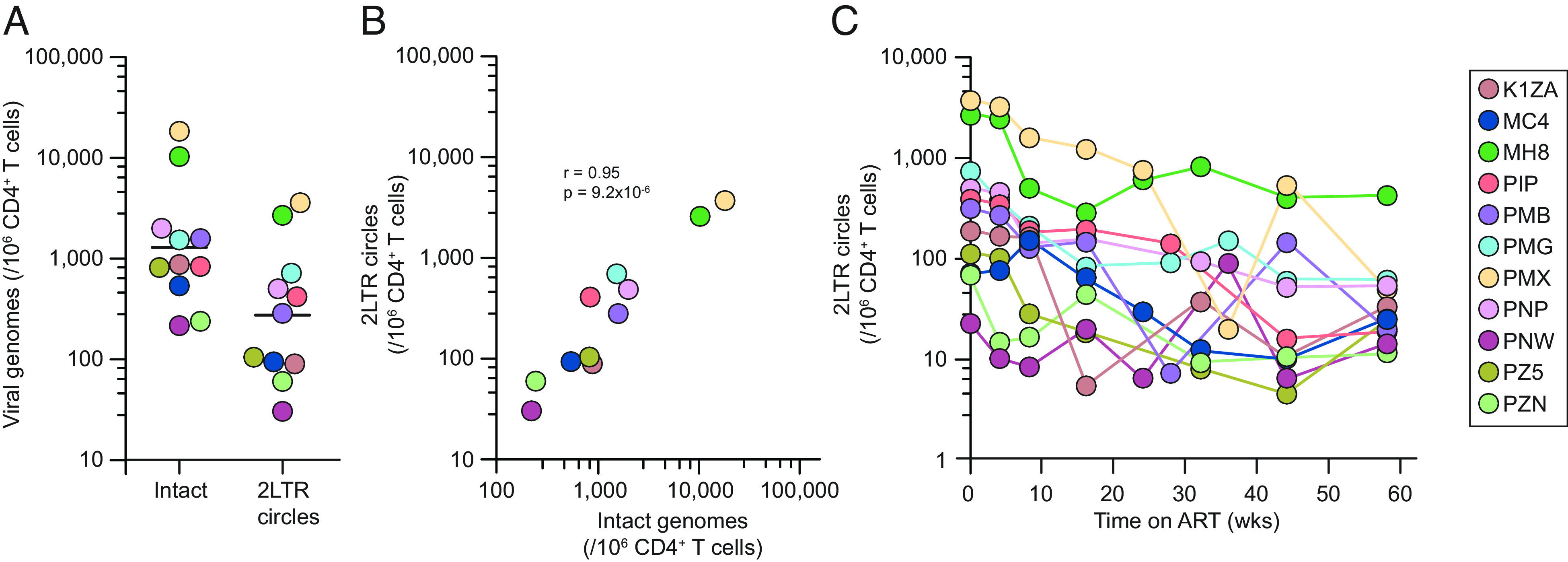
Decay of 2LTR circles. (*A*) Levels of 2LTR circles in peripheral blood CD4^+^ T cells at the time of ART initiation (week 0). Levels of intact genomes are shown for comparison. Values for intact genomes have been corrected for shearing and *env*^+^2LTR circles. The black line represents geometric mean. (*B*) Correlation between intact proviruses and 2LTR circles at the time of ART initiation. (*C*) Decay of 2LTR circles following ART initiation.

We also explored the dynamics of 2LTR circles. At the time of ART initiation, *env^+^*2LTR circles were detectable in all animals with a geometric mean frequency of 274 copies/10^6^ CD4^+^ T cells (Fig. 4*A*). This is approximately one fifth the level of intact genomes in the same samples (Fig. 4*A*). These results highlight the importance of correcting for 2LTR circles in PCR-based reservoir measurements in the SHIV model. As expected, the frequency of 2LTR circles is strongly correlated with the level of intact proviruses at the time of ART initiation (r = 0.95, Fig. 4*B*). Following initiation of ART, 2LTR circles declined in a biphasic fashion that resembled the decay of intact SHIV genomes (Fig. 4*C*) and the decay of HIV-1 and SIV 2LTR circles (47, 48). Because of the limited capacity of 2LTR circles to support viral gene expression, this decay may instead reflect normal contraction phase turnover of previously activated CD4^+^ T cells. 2LTR circles remained readily detectable at 10 to 100 copies/10^6^ CD4^+^ T cells even after a year (Fig. 4*C*). It is also possible that the decrease in the frequency of cells with 2LTR circles is due to dilution by proliferation of CD4^+^ T cells. Despite the unclear factors leading to the high levels of 2LTR circles in SHIV-infected rhesus macaques, it is crucial to exclude them from IPDA and other PCR-based reservoir measurements.

### Comparative Analysis of the Reservoir Sizes and Decay Dynamics in SIV, SHIV, and HIV-1.

Delineating similarities and differences between the SIV and SHIV reservoirs in relation to HIV-1 is critical for understanding the relevance of these models and for making accurate conclusions from preclinical studies. Near-full genome sequencing data have previously shed light on qualitative differences in the landscape of proviral genomes in ART-treated SIV, SHIV, and HIV-1 infections (49–58, 74). Recently, Fray et al. examined the decay kinetics of CD4^+^ T cells carrying SIV genomes and reported multiphasic decay that was generally faster than that observed in HIV-1 infection (47, 48). However, a comparative quantitative analysis of the decay of intact SHIV genomes relative to SIV and HIV-1 has not yet been described. Therefore, we compared the population average decay rates of intact SHIV genomes following ART initiation to those observed in previous studies of SIV and HIV-1 infection (47, 48).

**Fig. 5.**
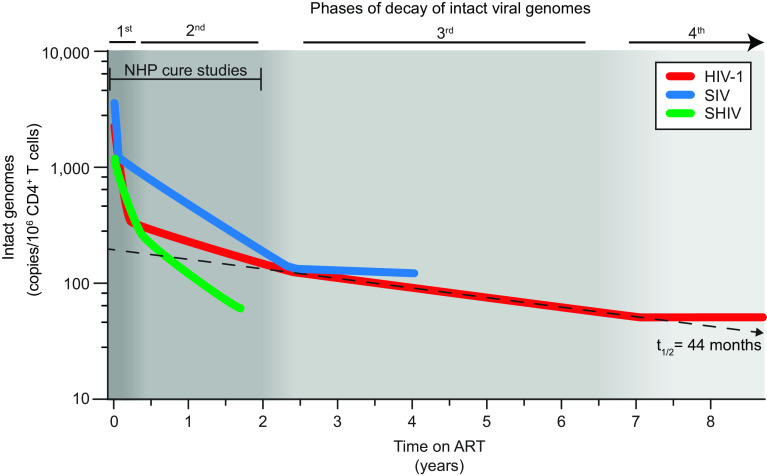
Summary of decay characteristics of intact HIV-1, SIV, and SHIV genomes following initiation of ART. Based on measurements made in circulating CD4^+^ T cells with the relevant IPDAs. The dashed line indicates decay with a t_1/2_ of 44 mo, as observed in PWH on long-term ART. Shading indicates distinct phases of decay. For HIV-1, the 4th phase may represent an increase in infected cell frequency (15). See *SI Appendix*, Table S3 for decay parameters and text for references.

As shown in Fig. 5, the initial decay of intact SHIV genomes in peripheral blood CD4^+^ T cells (t_1/2_ = 30.1 d or 4.3 wk) was slower than the initial decay of intact SIV and HIV-1 genomes (t_1/2_ = 3.4 d and 12.9 d, respectively). Interestingly, as shown in Fig. 5, the half-life of the next phase (t_1/2_ = 8.06 mo) was similar to that observed in SIV-infected macaques (t_1/2_ = 8.1 mo ref. 48), but faster than the second phase decay of intact HIV-1 genomes (t_1/2_ = 19 mo, ref. 47). In SIV-infected macaques, this second phase decay continues until approximately 2.3 y after ART initiation when a very stable third population of infected cells becomes dominant (48). This third phase population is likely similar to the stable population of latently infected cells (t_1/2_ = 44 mo, refs. 13 and 14) that constitutes the major barrier to curing HIV-1 infection. Interestingly, it is now clear that for HIV-1 infection there is a 4th phase in which infected cell frequencies may actually increase due to infected cell proliferation (15). Future studies in SHIV-infected macaques on very long-term ART would be needed to determine whether and when 3rd and 4th phases of decay occur in treated SHIV infection. In any event, the significance of these kinetic studies lies in the fact that most trials of cure interventions in SIV- and SHIV-infected macaques are carried out in animals that have been on ART for less than 2 y. During this period, the dominant populations of infected cells are decaying at a rate that is faster than that observed in PWH starting ART and much faster than the decay observed in PWH on long-term ART (Fig. 5). Because decay kinetics reflect the properties of distinct infected cell populations, it cannot be assumed that interventions would be equally effective against the stable populations of infected cells that persist in PWH on long-term ART.

### Use of Decay Rates in the Evaluation of Cure Interventions in SHIV-Infected Macaques.

**Fig. 6.**
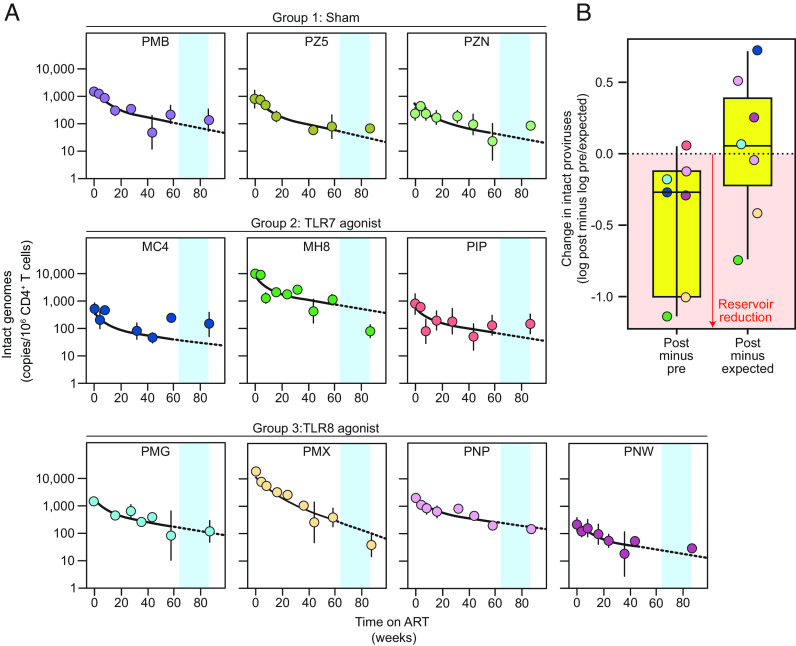
Effect of reservoir decay on evaluation of cure interventions. (*A*) Decay of intact SHIV genomes from the time of ART initiation through the period of TLR7/8 intervention (blue shading). The solid black line indicates biphasic decay models for individual animals determined using preintervention data. The dotted line indicates extension of predicted 2nd phase decay through the period of TLR7/8 agonist administration. (*B*) Evaluation of the efficacy of TLR7/8 administration using different metrics. Log scale differences in the frequency of intact viral genomes are shown for postintervention values minus preintervention values (*Left*) or postintervention values minus predicted values at that time point based on the rate of 2nd phase decay in each animal (*Right*). Negative values indicate a decrease in frequency.

Given that many cure studies in SHIV models are done before the quasi-stable 3rd phase of decay of intact viral genomes is reached (2.3 y in SIV-infected macaques, ref. 48), we examined how 2nd phase decay of intact SHIV genomes affects the evaluation cure interventions. This is important because there is significant natural decay during the 2nd phase (Fig. 5) that must be accounted for in the evaluation of reservoir-targeting interventions occurring during this period. A subset of the SHIV_SF162P3_-infected animals in our cohort were treated after 64 wk of ART with toll-like receptor (TLR) agonists that are currently being combined with therapeutic vaccines and HIV broadly neutralizing antibodies in clinical trials (37, 75, 76). The animals were randomly divided into three treatment groups: sham (group 1; n = 3), TLR7 agonist GS9620/Vesatolimod (group 2; n = 3), and TLR8 agonist GS720566 (group 3; n = 4). A parallel trial in SIV-infected macaques was carried out simultaneously and has been described in detail previously (77). SHIV-infected animals were administrated a total of 10 doses of the respective TLR agonists given every other week, with a 6-wk break between doses 6 and 7 (Fig. 2*A*). No significant toxicity was observed. Intact proviral decay curves extended through the intervention period are shown in Fig. 6*A*. IPDA analysis of reservoir size before and after the period of TLR7/8 agonist administration indicated a slight trend toward a decrease in the frequency of intact viral genomes (Fig. 6 *A* and *B*). To further understand whether treatment with TLR7/8 agonists impacted reservoir size, we predicted the expected levels of intact proviruses for each macaque at week 86 based on our fit of the biphasic decay model to the data obtained during the 64 wk of ART alone and compared them to values measured by the IPDA at week 86 (Fig. 6*B*). With this analysis, there was no apparent effect of the intervention. Thus, the efficacy of interventions should be analyzed in the context of the natural decay occurring over the period of the intervention and with robust, no-intervention control groups. Consistent with the lack of an intervention effect on the level of intact viral genomes, rapid viral rebound was observed in all three groups upon discontinuation of ART (*SI Appendix*, Fig. S2).

## Discussion

In preclinical trials of HIV-1 cure strategies, efficacy is ideally measured by a reduction in the number of cells constituting the stable latent reservoirs that represent the major barrier to cure. There are two main problems in making this measurement. First, the vast majority of proviruses persisting in the setting of ART are defective and unable to cause viral rebound (49–58). Standard single-amplicon DNA PCR assays drastically overestimate the size of the latent reservoir by detecting not only intact proviruses but also defective proviruses (61). Consequently, the use of such assays may obscure true changes to reservoir size if the intervention does not affect intact and defective proviruses equally. As a solution to this problem, we describe here a SHIV version of the IPDA, a multiplex ddPCR assay capable of better quantifying intact viral genomes by excluding proviruses with the most common fatal defects, namely large deletions and APOBEC3-mediated hypermutation (57, 58). Our analysis also excludes 2LTR circles, which are not competent for replication. We used this assay to address a second major problem in reservoir measurement, the presence in the period following ART initiation of an excess of intact viral genomes not destined to become part of the stable latent reservoir. We show here that intact SHIV genomes in circulating CD4^+^ T cells undergo biphasic decay during the first year of ART at rates considerably faster than reservoir decay in PWH on long-term ART (Figs. 3 and 5). The rapid first phase decay (t_1/2_ = 30.1 d) may reflect degradation of linear unintegrated forms in recently infected CD4^+^ T cells and/or the rapid turnover of infected CD4^+^ lymphoblasts (40, 44). The slower second phase decay (t_1/2_ = 8.1 mo) may reflect another subpopulation of infected CD4^+^ T cells whose properties are distinct from the extremely stable population of latently infected cells that persist in the setting of long-term ART. A significant problem with SIV and SHIV models uncovered here is that most interventions are tested during the period of 2nd phase decay. We show that the natural 2nd phase decay of intact SHIV genomes must be considered in the evaluation of interventions targeting the reservoir so that natural decay is not mistaken for an intervention-induced effect. In addition, for interventions tested during the period of 2nd phase decay, a caveat is that the intervention may not be equally effective in PWH on long-term ART whose reservoirs are dominated by latently infected cells with a slower decay rate. In classic models of viral dynamics, the multiphasic decay of plasma virus or infected cells is indicative of multiple distinct populations of infected cells (1). These populations may differ with respect to critical properties such as level of expression of viral genes and susceptibility to immune clearance.

Reservoir dynamics in HIV-1 infection were initially characterized using the QVOA (7–13). Before and immediately after ART initiation, the frequency of latently infected cells detected by QVOA is substantially higher than frequencies observed in PWH on long-term ART (43). These cells follow a biphasic pattern of decay, with an initial rapid decay in the first 3 mo of ART, followed by a much slower decay in the subsequent months. These findings gave rise to the notion that measurement of the latent reservoir should not be done during the first several months of ART because most of the viral genomes detected during that period are not destined to become part of the stable latent reservoir. However, detailed analysis of the initial decay kinetics of viral genomes has not been possible until recently. The QVOA provides a definitive minimal estimate of the frequency of cells with inducible replication-competent viral genomes. However, it is a labor-intensive assay requiring large blood samples and is therefore unsuitable for detailed kinetic studies. Simpler single amplicon PCR assays are also unsuitable because they mainly detect defective proviruses (57, 61). For these reasons, the IPDA has proven particularly useful in evaluating reservoir dynamics (46–48, 57, 63). It is noteworthy that these IPDA studies independently corroborated the very slow decay of latently infected cells determined by the QVOA in PWH on long-term ART. Furthermore, White et al. (47) used the IPDA to define the early decay kinetics of intact HIV-1 genomes and demonstrated two early phases of decay (t_1/2_ = 12.9 d and 19 mo) that occur before the quasi-stable 3rd phase (t_1/2_ = 44 mo) is reached. These results have important implications for understanding reservoir measurements in PWH who have been on ART for different lengths of time.

Due to the challenges associated with performing outgrowth assays with the small volumes of blood available in NHP studies, similar in-depth analyses of decay of intact viral genomes in monkeys were not performed until the development of an IPDA for SIV (48, 58). Fray et al. used the SIV IPDA to analyze decay kinetics in a cohort of 10 SIV_mac251_-infected rhesus macaques on ART for 4 y (48). They identified two initial phases of decay (t_1/2_ = 3.3 d and 19 mo) before a very stable 3rd phase became evident. Here, we used the SHIV IPDA to describe the decay kinetics of intact SHIV genomes during the first year of ART in 11 chronically infected rhesus macaques. As with HIV-1 and SIV, intact SHIV genomes also undergo initial biphasic decay (t_1/2_ = 30.1 d and 8.1 mo). A third and even a fourth phase have been reported in PWH on long-term ART (13–15, 46), and a 3rd phase has been described in SIVmac_251_-infected macaques (48). However, since the animals in the present SHIV cohort were not on ART beyond 90 wk, we could not address later phases directly. A summary of the different phases of decay for HIV-1, SIV, and SHIV is presented in Fig. 5 and *SI Appendix*, Table S3.

Interestingly, the first phase decay of intact SHIV genomes (t_1/2_ = 30.1 d) was considerably slower than the first phase decay of plasma virus (t_1/2_ < 3 d). According to the classic models of viral dynamics (1, 41, 42), the rapid decay of plasma virus following initiation of ART must reflect the decay of virus-producing cells. ART blocks new infection of susceptible cells, and the half-life of virions in the plasma is very short (minutes). Thus, the decay rate of plasma virus must reflect the decay of the population of infected cells that is responsible for producing most of the plasma virus. We did not find a population of infected CD4^+^ T cells in the circulation that had a rapid decay rate matching the decay of plasma virus. This suggests that most of the plasma virus is produced by infected cells in the tissues, a concept that is consistent with the fact that the activation events that render CD4^+^ T cells susceptible to infection occur in the secondary lymphoid organs. Confirmation is difficult because it requires repeated sampling of tissue sites at close time intervals after initiation of ART. One study of PWH on stable ART suggests that the frequency of CD4^+^ T cells with intact proviruses is similar in blood and lymph nodes (78).

Our analysis also included direct measurement of 2LTR circles. These circular forms of the genome are not competent for replication but have generated considerable interest as potential markers of new infection events, a notion that is based on the assumption that the circles decay quickly (68, 69). However, other circular DNA molecules such T cell receptor excision circles and herpesvirus genomes demonstrate considerable in vivo stability, and several studies have shown that HIV-1 and SIV 2LTR circles can also be stable provided that the host cell is not eliminated (47, 48, 66, 67). Here, we show that in SHIV infection, 2LTR circles undergo biphasic decay during the first year of ART but remain readily detectable. Thus, they should be excluded from reservoir measurements as described here.

Versions of the SHIV IPDA have been used in recent reservoir studies (79). Here, we used the SHIV IPDA to track changes in the frequency of intact proviruses before and after treatment with TLR7/8 agonists. With their potential to indirectly induce HIV-1 expression in CD4^+^ T cells and modulate a variety of immune effector mechanisms, TLR agonists have garnered significant interest in the HIV-1 cure field (37, 75, 76). The TLR7 agonist GS-9620 (Vesatolimod) has been tested as a latency-reversing agent in several NHP models (37, 39, 76). We show here that simple before and after comparisons of reservoir size may not be sufficient to judge the efficacy of shock-and-kill interventions if significant natural decay is occurring during the period of the intervention. A consideration of the trajectory of natural 2nd phase decay for each animal and the inclusion of adequately sized control groups are essential.

Our study has several limitations. The decay rates described in this study were determined in a chronic infection model, and different rates may be observed in animals that are treated with ART shortly after infection. In addition, the decay rates observed with SHIVSF162P3 may not be representative of all SHIV variants, and it will be important to determine whether similar rates are observed in SHIV strains that replicate more robustly and induce intestinal immunopathology (80, 81). All analyses were confined to peripheral blood CD4^+^ T cells and thus exclude tissue-resident cells not found in circulation. Interestingly, for HIV-1, SIV, and SHIV, the decay of plasma virus following initiation of ART is very rapid, with an initial phase t_1/2_ of ~1 d (1, 16, 23, 41, 42, 48, 71). The initial decay of intact HIV-1, SIV, and SHIV genomes in circulating CD4^+^ T cells is much slower (t_1/2_ = 12.1 d, 3.3 d, and 30.1 d, respectively; *SI Appendix*, Table S3). This suggests that the cells producing most of the plasma virus are localized to the lymphoid tissues. As with all versions of the IPDA, it is important to note that not all double-positive genomes detected by the assay represent readily inducible replication-competent proviruses. Some replication-competent HIV-1 proviruses are induced only after multiple rounds of global T cell activation in the QVOA (42, 59, 60). In addition, some double-positive proviruses contain minor defects that affect viral fitness (57), and sequence polymorphisms can affect amplification in the IPDA (82). We did not observe amplification failures related to sequence polymorphisms in the current study. Such events would be readily apparent as the absence of any signal from one of the IDPA amplicons and would require the use of alternative primers or probes (39).

In summary, we have developed an IPDA for SHIV and used it to define the early decay kinetics of intact SHIV genomes. By characterizing the frequency and decay rate of intact SHIV genomes during the first year of ART, we have provided a picture of the natural decay that occurs in the absence of additional intervention. Future cure studies using the SHIV model should consider these reservoir dynamics and account for them in study design.

## Methods

### Animals and Study Design.

Eleven outbred, Indian-origin female rhesus macaques *(Macaca mulatta)* negative for the protective alleles Mamu-A*01, Mamu-B*08, and Mamu-B*17 were challenged repeatedly with intrarectal inoculations of a SHIV_SF162P3_ stock. Plasma virus levels were measured every week, and the animals were considered infected if viremic for 2 wk. At week 89 after the initial inoculation, all animals were treated with a suppressive combination ART regimen of dolutegravir, tenofovir disoproxil fumarate, and emtricitabine (DTG/TDF/FTC) (22). Blood samples were collected at multiple time points, and peripheral blood mononuclear cells (PBMCs) were viably cryopreserved. All animals were housed at Bioqual Inc. in Rockville, MD and maintained in accordance with the guidelines in the Guide for the Care & Use of Laboratory Animals, 8th Edition (NIH). All monkey studies were approved by the Institutional Animal Care & Use Committees at Beth Israel Deaconess Medical Center and Bioqual.

### TLR7/8 Agonist Treatment.

Animals were randomly divided into three treatment groups: sham (group 1; n = 4), TLR7 agonist GS9620/Vesatolimod (group 2; n = 3), and TLR8 GS720566 agonist (group 3; n = 3). Beginning at 64 wk after ART initiation, agonists were administrated in 10 doses given every other week, with a 6-wk break between doses 6 and 7. Details of the treatment protocol have been previously described for a parallel study in SIV-infected animals (75). The last dose was injected at week 86. ART was discontinued at week 90, and viral rebound was monitored by frequently sampling plasma until day 56 after ART discontinuation.

### Isolation of Macaque CD4^+^ T Lymphocytes.

PBMCs previously obtained from whole blood and viably cryopreserved in liquid nitrogen were thawed in RPMI with 20% heat-inactivated FBS. Untouched total CD4^+^ T cells were isolated using negative depletion (CD4 T Cell Isolation Kit, NHP, Miltenyi Biotec). Following isolation, up to 5 × 10^6^ CD4^+^ T cells were pelleted and resuspended for DNA extraction.

### DNA Isolation.

Genomic DNA was isolated from total CD4^+^ T cells using the QIAamp DNA Mini kit (Qiagen). DNA concentration was measured using NanoDrop 2000 and/or Qubit 4 Fluorometer with the Qubit dsDNA BR Assay Kit (ThermoFisher Scientific).

### Viral RNA Measurement.

Viral RNA was isolated from cell-free plasma using a viral RNA extraction kit (Qiagen) and quantified essentially as previously described (27).

### SHIV IPDA.

Intact SHIV genomes were quantified using a method similar to the previously published HIV-1 IPDA (57) and SIV IPDA (58). Briefly, 5.5 μL of genomic DNA were added to 16.5 μL of a mastermix containing 10 μL of 2× ddPCR Supermix for Probes (no dUTP, Bio-Rad), 600 nM of each primer, and 200 nM of each probe, in a total reaction volume of 22 μL. Droplets were made using the Bio-Rad QX200 Manual DG ddPCR system with the appropriate manufacturer-supplied consumables. Droplets underwent thermal cycling as follows: 10 min at 95 °C, 50 cycles of 30 s at 95 °C and 2 min at 56 °C, and 10 min at 98 °C with a ramp rate of 2 °C per second. The thermal-cycled droplets were read on a QX200 Droplet Reader (Bio-Rad). Output data were analyzed using QuantaSoft Studio Software (Bio-Rad). Intact genomes per million CD4^+^ T cells are reported after subtracting 2LTR circles with intact *env* and correcting for DNA shearing as previously described for SIV (58). Primer and probe sequences are given in *SI Appendix*, Table S4.

We also tested whether the SHIV IPDA designed using SHIV_SF162P3_ sequences could be applied to other commonly used SHIVs. We found that all primers and probes match the consensus sequence for SHIV_AD8_, another widely used CCR5-tropic SHIV (34, 80). We found a single nucleotide polymorphism in both forward primers for SHIV_CH505_ (81). After modifying the forward primers, we used the SHIV_CH505_-adapted version of the IPDA to quantify the frequency of intact proviruses in a cohort of infant rhesus macaques infected with SHIV_CH505_ (79). Thus, the SHIV IPDA can readily be adapted for quantifying intact proviruses in macaques infected with different SHIVs.

### *env*-2LTR Circle Duplex Assay.

2LTR circles were measured using the SIV 2LTR primers and probe adapted from Policicchio et al. (70) duplexed with the SHIV IPDA *env* amplicon. As described above, 5.5 µL of DNA were added to 16.5 µL master mix containing 10 μL of 2× ddPCR Supermix for Probes (no dUTP) (Bio-Rad), primers at a final concentration of 600 nM, and probes at a final concentration of 200 nM, in a total reaction volume of 22 µL.

### RPP30 ddPCR Assay.

Simultaneous quantification of DNA shearing and input cell equivalents was carried out by measuring two amplicons in the Rhesus macaque RPP30 gene spaced at the same distance as the SHIV IPDA amplicons. For each 22 µL reaction, up to 3 ng of DNA was added to 16.5 µL of mastermix consisting of 2× ddPCR Supermix for Probes (no dUTP) (Bio-Rad) and 1.1 µL of assay premix of primers and probes for each amplicon (IDT), such that primers were at a final concentration of 500 nM, and probes, 250 nM. Outputs of the IPDA and the 2LTRc ddPCR reactions were normalized to copies per million cells using the cell equivalents calculated from the RPP30 reaction. A DSI was determined as previously described (57). This index was applied to correct for shearing that could reduce the number of double-positive IPDA events. Primers and probes are given in *SI Appendix*, Table S4.

## Mixed-Effect Modeling

A non-linear mixed-effect approach was used to fit the decay of intact SHIV DNA in infected macaques on ART. The general model is governed by the following equation:

$$Y=Y_{0}(Ae^{-b_{1}t}+\left(1-A\right)e^{{-b}_{2}t}),$$

where *Y* represents the amount of intact SHIV DNA (copies/10^6^ cells), *Y_0_* is the baseline value, *A* is the fraction of *Y* that decays in the first phase with decay rate *b_1_*, and (1−*A*) is the fraction of *Y* that decays in the second phase with decay rate *b_2_*.

This general model allows one to test multiple models of interest. By fixing *A* = 1, we can test for a single exponential decay model, estimating only *b_1_* without estimating *b_2_*. Model comparison was done using the corrected Bayesian Information Criterion (BICc) that penalizes the quality of the fit by the number of model parameters estimated. The model with the lowest BICc is preferred. We found that the biexponential decay model showed a lower BICc (302) compared to a single decay model (BICc = 306), despite estimating two extra parameters.

The models were fit to the data from all 11 SHIV-infected animals simultaneously and parameters were estimated using the Monolix software version 2023R1 (Lixoft) (https://doi.org/10.1201/b17203). The best model fit was selected from 100 different rounds of parameter estimation with randomly chosen initial parameter values. Based on the Fisher Information Matrix, 95% CIs were calculated using the Monolix function *confintmlx.*

## Supplementary Material

Appendix 01 (PDF)

## Data Availability

All study data are included in the article and/or *SI Appendix*.
