# Supplementary material for: Biphasic decay of intact SHIV genomes following initiation of antiretroviral therapy complicates analysis of interventions targeting the reservoir
Source: Proc Natl Acad Sci U S A. 2023 Oct 16;120(43):e2313209120. doi: 10.1073/pnas.2313209120 (PMC10614214; doi:10.1073/pnas.2313209120)
Supplement: Supplementary file 1 — Appendix 01 (PDF) [file pnas.2313209120.sapp.pdf]

Supplemental Information for:

Biphasic decay of intact SHIV genomes following initiation of antiretroviral therapy complicates analysis of interventions targeting the reservoir

Mithra Kumar[1*], Emily J. Fray[1*], Alexandra M. Bender[1], Carolin Zitzmann[2], Ruy Ribeiro[2], Alan S. Perelson[2], Dan H. Barouch[3], Janet D. Siliciano[1], Robert F. Siliciano[1,4].

Figure S1. DNA shearing index (DSI)
Figure S2. Rebound kinetics

Table S1. Estimated population parameters for intact SHIV DNA decay under ART
Table S2. Estimated individual parameters for intact SHIV DNA decay under ART
Table S3. Comparison of decay kinetics of intact viral genomes for HIV-1, SIV, and SHIV.
Table S4. SHIV IPDA primers and probes.

A B

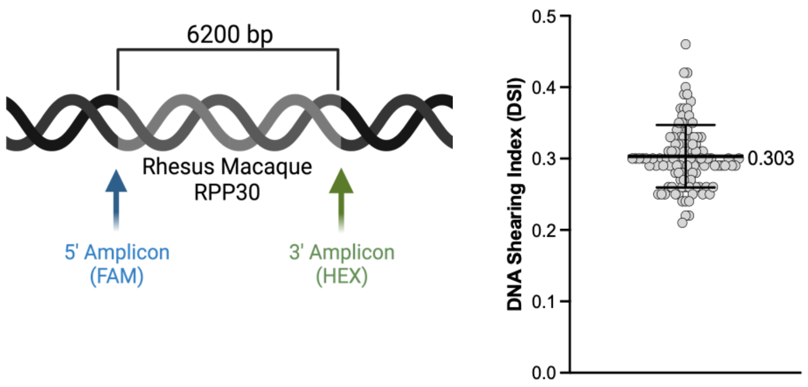

Fig. S1. Determination of DNA shearing and input cell equivalents by ddPCR. (**A**) ddPCR amplification of two regions of the macaque RPP30 gene spaces at ~6200 nucleotides apart. This is the same spacing as the *gag* and *env* amplicons in the SHIV IPDA (Fig. 1A), allowing for calculation of a DNA shearing index (DSI) as previously described (57) as well as determination of input cell equivalents. (**B**) Distribution of DSI values for the SHIV-infected macaques analyzed in this study.

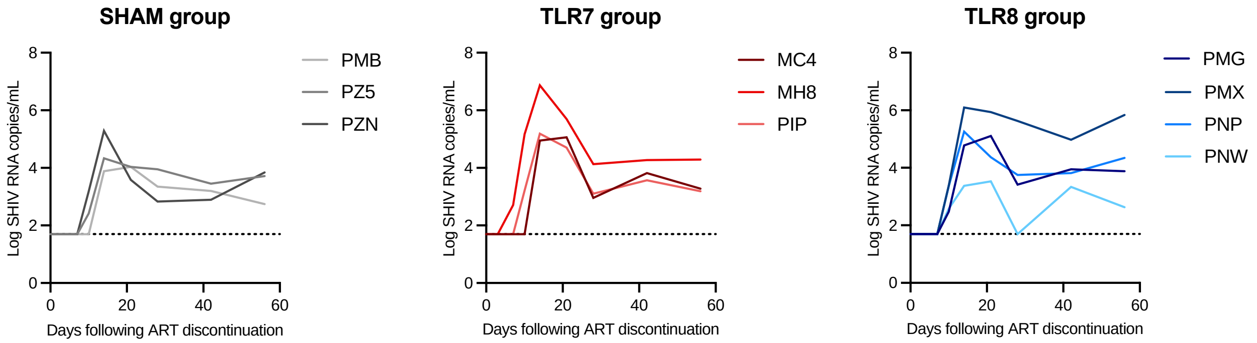

Figure S2. Rebound kinetics for animals in each treatment group.

TABLE S1. Estimated population parameters for intact SHIV DNA decay under ART*

	Log (Y_0)	A	b_1 (w^{-1})	b_2 (w^{-1})	$t_{1/2}$ 1st (w)	$t_{1/2}$ 2nd (w)
Estimate	3.08	0.71	0.16	0.019	4.3	35
95% CI	2.77-3.42	0.27-0.94	0.045-0.44	0.0064-0.058	1.6- 15	12-108

*A non-linear mixed effect approach was used to fit the decay of intact SHIV genomes. The general model is governed by the following equation:

$$Y = Y_0(Ae^{-b_1 t} + (1 - A)e^{-b_2 t})$$

where Y represents the amount of intact SHIV DNA (copies/10^6 cells), Y_0 is the baseline value, A is the fraction of Y that decays in the first phase with decay rate b_1, and (1-A) is the fraction of Y that decays in the second phase with decay rate b_2. Parameter estimates for individual animals are given in **Table S2**.

TABLE 2. Estimated individual parameters for intact SHIV DNA decay under ART*

ID	Y_0	*A*	b_1	$t_{½}$ (wks)	b_2	$t_{½}$ (wks)
K1ZA	2.88	0.71	0.198	3.5	0.017	40.5
MC4	2.62	0.73	0.180	3.9	0.017	41.9
MH8	3.91	0.71	0.184	3.8	0.018	38.2
PIP	2.87	0.71	0.232	3.0	0.018	39.3
PMB	3.18	0.72	0.151	4.6	0.022	31.2
PMG	3.29	0.71	0.155	4.5	0.019	36.1
PMX	4.06	0.72	0.140	4.9	0.040	17.4
PNP	3.36	0.70	0.176	3.9	0.016	42.7
PNW	2.45	0.71	0.156	4.4	0.019	36.8
PZ5	2.94	0.72	0.152	4.5	0.024	28.7
PZN	2.72	0.71	0.142	4.9	0.021	32.4

*See **Table S1** for explanation of symbols and modeling method.

Table S3. Comparison of decay kinetics of intact viral genomes for HIV-1, SIV, and SHIV.

Virus	1st phase*		2nd phase		3rd phase		4th phase‡	References
	$t_{1/2}$ (d)	Transition to next phase† (mo)	$t_{1/2}$ (mo)	Transition to next phase† (yrs)	$t_{1/2}$ (mo)	Transition to next phase† (yrs)	t_2 (yrs)	
HIV-1	12.9	3-4	19	2-3	44	3-7	23	13-15, 46, 47
SIV	3.3	1	8.1	2.3	∞	ND§	ND	48
SHIV	30.1	3-5	8.1	ND	ND	ND	ND	This study

*Refers to the first phase decay of intact viral genomes measured by IPDA or QVOA in circulating $CD4^+$ T cells. Note that the first phase decay of plasma virus is faster.

†Approximate average time on ART after which the next phase of decay become apparent.

‡Because QVOA studies indicate an increase in the frequency of latently infected cells after 7 years of ART, a doubling time (t_2) is given.

§ND, not determined. Longer studies will be needed to determine parameters for 3rs and 4th phase decay on SIV and SHIV proviruses.

Table S4. SHIV IPDA primers and probes.

Amplicon	Primer/Probe Name	Sequence	Fluorophore	Quencher
$SHIV_{SF162P3}$ IPDA *gag*	gagF	CAC TCT GCA AGG CAA TGC A	N/A	N/A
	gagR	GGG CAT TTG GCC ATA ACA TG	N/A	N/A
	gag intact probe	TTC CAC ATT TCC AGC AT	FAM	MGBNFQ
	gag HM probe	TTT TCT ACA TTT CTA GCA T	N/A	MGBNFQ
$SHIV_{SF162P3}$ IPDA *env*	envF	AGT GGT GCA GAG AGA AAA AAG AGC	N/A	N/A
	envR	GTC TGG CCT GTA CCG TCA GC	N/A	N/A
	env intact probe	CCT TGG GTT CTT GGG A	VIC	MGBNFQ
	env HM probe	CCT TAG GTT CTT AGG AGC	N/A	MGBNFQ
$SIVmac_{251}$ 2LTRc	2LTRc F	CGC CTG GTC AAC TCG GTA CTC	N/A	N/A
	2LTRc R	GGT ATG ATG CCT TCT TCC TTT TCT AAG	N/A	N/A
	2LTRc probe	CCC TGG TCT GTT AGG ACC CTT TCT GCT TTG	FAM	MGBNFQ*
Rhesus RPP30-1	RPP30-1F	CAG CAT GAT TGT CTC AGC	N/A	N/A
	RPP30-1R	AAT TCA TAA AGG CTC TCA AG	N/A	N/A
	RPP30-1 probe	ATC AGC CAT TTG CTA GTA TAA TCC CTC	FAM	ZEN/IABkFQ
Rhesus RPP30-2	RPP30-2F	ACC TGC TCA TTA GAC TTG ATA GGA	N/A	N/A
	RPP30-2R	CAT GGT CCA TTT ATC CCA CAA AC	N/A	N/A
	RPP30-2 probe	AGC TCT GGG ACA ATT CTT GGC ACA	HEX	ZEN/IABkFQ
